# Fractional Order Two-Temperature Dual-Phase-Lag Thermoelasticity with Variable Thermal Conductivity

**DOI:** 10.1155/2014/646049

**Published:** 2014-10-28

**Authors:** Sudip Mondal, Sadek Hossain Mallik, M. Kanoria

**Affiliations:** ^1^Bhatkunda High School, Burdwan 713153, India; ^2^Department of Mathematics, Aliah University, Kolkata 700091, India; ^3^Department of Applied Mathematics, University of Calcutta, Kolkata 700009, India

## Abstract

A new theory of two-temperature generalized thermoelasticity is constructed in the context of a new consideration of dual-phase-lag heat conduction with fractional orders. The theory is then adopted to study thermoelastic interaction in an isotropic homogenous semi-infinite generalized thermoelastic solids with variable thermal conductivity whose boundary is subjected to thermal and mechanical loading. The basic equations of the problem have been written in the form of a vector-matrix differential equation in the Laplace transform domain, which is then solved by using a state space approach. The inversion of Laplace transforms is computed numerically using the method of Fourier series expansion technique. The numerical estimates of the quantities of physical interest are obtained and depicted graphically. Some comparisons of the thermophysical quantities are shown in figures to study the effects of the variable thermal conductivity, temperature discrepancy, and the fractional order parameter.

## 1. Introduction

During the last five decades, nonclassical thermoelasticity theories involving hyperbolic type heat transport equations admitting finite speed of thermal signals have been formulated. According to these theories, heat propagation is to be viewed as a wave phenomenon rather than a diffusion phenomenon.

In order to overcome the paradox of an infinite speed of thermal wave inherent in CTE and CCTE (classical coupled theory of thermoelasticity), efforts were made to modify coupled thermoelasticity, on different grounds, to obtain a wave-type heat conduction equation by different researchers. Lord and Shulman [1] formulated the generalized thermoelasticity theory introducing one relaxation time in Fourier's law of heat conduction equation and thus transforming the heat conduction equation into a hyperbolic type.

Green and Lindsay [2] used the theory of two different relaxation times in the constitutive relations for the stress tensor and the entropy equation. Later Green and Naghdi [3–5] have proposed three models, labeled as types I, II, and III. When they are linearized, type I is the same as the classical heat equation (based on Fourier's law) whereas the linearized versions of type-II and type-III theories permit propagation of thermal waves at finite speed. The entropy flux vector in type-II (i.e., thermoelasticity without energy dissipation) and type-III (i.e., thermoelasticity with energy dissipation) models is determined in terms of the potential that also determines stresses. When Fourier conductivity is dominant, then the temperature equation reduces to classical Fourier's law of heat conduction and when the effect of conductivity is negligible, then the equation has undamped thermal wave solutions without energy dissipation.

Tzou [6] introduced two-phase-lag models to both the heat flux vector and the temperature gradient. According to this model, classical Fourier's law $\vec{q}=-K\vec{\nabla }\theta$ has been replaced by $\vec{q}(P,t+\tau_{q})=-K\nabla \theta (P,t+\tau_{T})$, where the temperature gradient $\vec{\nabla }\theta$ at a point *P* of the material at time *t* + *τ*_*T*_ corresponds to the heat flux vector $\vec{q}$ at the same point at time *t* + *τ*_*q*_. Here *K* is the thermal conductivity of the material. The delay time *τ*_*T*_ is interpreted as that caused by the microstructural interactions and is called the phase-lag of the temperature gradient. The other delay time *τ*_*q*_ is interpreted as the relaxation time due to the fast transient effects of thermal inertia and is called the phase-lag of the heat flux. The case *τ*_*q*_ = *τ*_*T*_ = 0 corresponds to classical Fourier's law. If *τ*_*q*_ = *τ* and *τ*_*T*_ = 0, Tzou refers to the model as single-phase-lag model. Recently Several researchers have attempted to solve their problems on the basis of the theory of dual-phase-lag model. Roychoudhuri [7] has studied one-dimensional thermoelastic wave propagation in an elastic half-space in the context of dual-phase-lag model. The exponential stability [8] and condition of the delay parameters in the dual-phase-lag theory [9] under this model have been studied by Quintanilla. Wang et al. [10, 11] have studied the well-posedness and solution structure of the dual-phase-lag heat conduction equation. Wang and Mingtian [12] have studied the thermal oscillation and resonance in dual-phase-lag heat conduction equation. Ailawalia and Budhiraja have studied a problem of dual-phase-lag model with internal heat source [13].

Gurtin and Williams [14, 15] have suggested that there is no* a priori* ground for assuming that the second law of thermodynamics for continuous bodies involves only a single temperature; that is, it is more logical to assume a second law in which the entropy contribution due to heat conduction is governed by one temperature, that of the heat supply by another.

Chen and Gurtin [16] and Chen et al. [17, 18] have formulated a theory of heat conduction in deformable bodies, which depends on two distinct temperatures: the conductive temperature *ϕ* and the thermodynamic temperature *θ*. For time-independent situations, the differences between these two temperatures are proportional to the heat supply, and in the absence of any heat supply, the two temperatures are identical [18]. For time-dependent problems, however, and for wave propagation problems in particular, the two temperatures are, in general, different, independent of the presence of a heat supply. The key element that sets the two-temperature thermoelasticity (2TT) apart from the classical theory of thermoelasticity (CTE) is the material parameter *a*(≥)0, called the temperature discrepancy [18]. Specifically, if *a* = 0, then *ϕ* = *θ* and the field equations of the 2TT reduce to those of CTE.

The linearized version of the two-temperature theory (2TT) has been studied by many authors. Warren and Chen [19] have investigated the wave propagation in the two-temperature theory of thermoelasticity. Lesan [20] has established uniqueness and reciprocity theorems for the 2TT. Puri and Jordan [21] have studied propagation of plane waves under the 2TT. The existence, structural stability, and spatial behavior of the solution in 2TT have been discussed by Quintanilla [22].

It should be pointed out that both CTE and 2TT suffer from the so-called paradox of heat conduction, that is, the prediction that a thermal disturbance at some point in a body is felt instantly, but unequally, throughout the body. Mondal et al. [23, 24] have studied problems on two-temperature Green Naghdi III and dual-phase-lag model with variable thermal conductivity. Pal et al. [25], Islam et al. [26], Das and Kanoria [27], and Banik and Kanoria [28] have studied on two-temperature generalized thermoelasticity. Kumar et al. [29, 30] have established variational and reciprocal principles and some theorems in two-temperature generalized thermoelasticity. Recently Ailawalia et al. have solved a dynamic problem on Green-Naghdi (Type III) half-space with two-temperature [31] theory.

In this work, we have studied the thermoelastic stress, strain, displacement, conductive temperature, and the thermodynamic temperature in an infinite, isotropic, homogeneous elastic half-space under thermal shock using two-temperature dual-phase-lag generalized thermoelasticity in the context of fractional heat conduction equation. The governing equations of two-temperature generalized thermoelasticity theory are formed in the Laplace transform domain which are then solved by state space approach. The inversion of the transformed solutions is carried out numerically, applying a method based on a Fourier series expansion technique [32]. Finally, numerical estimate for different thermophysical quantities are obtained for copper materials. A complete and comprehensive analysis of the results is presented and the effects of fractional order parameter, two-type temperature, and variable thermal conductivity are discussed.

## 2. Development of Fractional Order Theory 

Differential equations of fractional order have been the focus of many studies due to their frequent appearance in various applications in fluid mechanics, viscoelasticity, biology, physics, and engineering. The most important advantage of using fractional differential equations in these and other applications is their nonlocal property. It is well-known that the integer order differential operator is a local operator but the fractional order differential operator is nonlocal. This means that the next state of a system depends not only upon its current state, but also upon all of its historical states. This is more realistic, and this is one reason why fractional calculus has become more and more popular [33–35].

Fractional calculus has been used successfully to modify many existing models of physical processes. One can state that the whole theory of fractional derivatives and integrals was established in the second half of the nineteenth century. The first application of fractional derivatives was given by Abel who applied fractional calculus in the solution of an integral equation that arises in the formulation of the tautochrone problem. The generalization of the concept of derivative and integral to a noninteger order has been subjected to several approaches, and some various alternative definitions of fractional derivatives have appeared [36–39]. In the last few years, fractional calculus has been applied successfully in various areas to modify many existing models of physical processes, for example, chemistry, biology, modeling and identification, electronics, wave propagation, and viscoelasticity. One can refer to Podlubny [35] for a survey of applications of fractional calculus.

Youssef [40] introduced the formula of heat conduction given by

(1) $$\begin{array}{c} q_{i}+\tau_{0}\frac{\partial q_{i}}{\partial t}=-KI^{\xi -1}\theta {,}_{i},\;0<\xi \leq 2, \end{array}$$

where the notation *I* ^*ξ*^ is the Riemann-Liouville fractional integral, introduced as a natural generalization of the well-known *n*-fold repeated integral *I* ^*n*^ *f*(*t*) written in a convolution-type form as in [41] which is written as follows:

(2) Inf(t)=1Γ(n)∫0t(t−τ)n−1f(τ)dτ, 0<n≤2,=f(t), n=0,

where Γ(*n*) is the Gamma function. A uniqueness theorem also has been proved. Sur and Kanoria have employed the theory to study problems on functionally graded [42] and viscoelastic material [43].

Ezzat and El-Karamany [44, 45] established a new model of fractional heat conduction equation by using the new Taylor series expansion of time-fractional order, developed by Jumarie [46] as

(3) $$\begin{array}{c} q_{i}+\frac{\tau_{0}^{\xi }}{\xi !}\frac{\partial^{\xi }q_{i}}{\partial t^{\xi }}=-K\theta {,}_{i},\;0<\xi \leq 1. \end{array}$$

El-Karamany and Ezzat [47] introduced two general models of fractional heat conduction law for a nonhomogeneous anisotropic elastic solid. Uniqueness and reciprocal theorems are proved, and the convolutional variational principle is established and used to prove a uniqueness theorem with no restriction on the elasticity or thermal conductivity tensors except symmetry conditions. For fractional thermoelasticity not involving two-temperature, El-Karamany and Ezzat [48] established the uniqueness, reciprocal theorems and convolution variational principle. For two-temperature theory the formula of heat conduction has been replaced by

(4) $$\begin{array}{c} q_{i}+\tau_{0}\frac{\partial q_{i}}{\partial t}=-KI^{\xi -1}\phi {,}_{i},\;0<\xi \leq 2, \end{array}$$

where *ϕ* is the conductive temperature. Two-temperature fractional order thermoelasticity problem of LS and Green Naghdi models (types II and III) have been solved by Sur and Kanoria [49].

Several researchers have solved different problems [50–54] using fractional order generalized thermoelasticity theory. More detailed discussion on the subject is available in the books of Hetnarski and Eslami [55], Eslami et al. [56], and Ignaczak and Ostoja-Starzewski [57].

## 3. Basic Formulation

The constitutive equations are

(5) $$\begin{array}{c} \sigma_{ij}=2\mu e_{ij}+\left(\lambda e-\gamma \theta \right)\delta_{ij},\;i,j=1,2,3, \end{array}$$

where *e*_*ij*_ = (1/2)(*u*_*i*,*j*_ + *u*_*j*,*i*_) and *e* = *e*_*kk*_.

In the context of two-temperature dual-phase-lag (2TDPL) generalized thermoelasticity theory, the equation of motion in the absence of body forces and the heat conduction equation in absence of heat sources for a linearly isotropic generalized thermoelastic solid based on the theory of fractional integral are, respectively, given by [58]

(6) $$\begin{array}{c} \rho {\ddot{u}}_{i}=\left(\lambda +\mu \right)u_{j,ji}+\mu u_{i,jj}-\gamma \theta_{,i},\;i,j=1,2,3, \end{array}$$

(7) [KIξ−1(1+τT∂∂t+τT22∂2∂t2)ϕ,i],i =(1+τq∂∂t+τq22∂2∂t2)(Kκθ˙+γT0e˙),

where *ρ* is the density, *λ* and *μ* are Lamé's constants, *K* is thermal conductivity, *γ* = (3*λ* + 2*μ*)*α*_*t*_,  *α*_*t*_ being the coefficient of linear thermal expansion, *T*_0_ is the reference temperature, and *κ* = *K*/*ρc*_*E*_,  *c*_*E*_ being the specific heat at constant strain.

The relation between conductive temperature (*ϕ*) and thermodynamic temperature (*θ*) is given by

(8) $$\begin{array}{c} \phi -\theta =a\phi_{,ii}\;i=1,2,3, \end{array}$$

where *a*(≥0) is the two-temperature parameter, called temperature discrepancy.

We will consider the thermal conductivity as a linear function of thermodynamical temperature as follows [59]:

(9) $$\begin{array}{c} K\left(\theta \right)=K_{0}\left[1+K_{1}\theta \right], \end{array}$$

where *K*_0_ is a constant which is equal to the thermal conductivity of the material when it does not depend on thermodynamical temperature (*θ*) and *K*_1_ is a nonpositive small parameter.

Substituting from (8) into (9), we get

(10) K(θ)=K0[1+K1ϕ−aK1ϕ,ii]or K(θ)=K0[1+K1ϕ]−aK0K1ϕ,iior K(θ)=K(ϕ)−aK0K1ϕ,ii.

We now use the following mapping [59]:

(11) ϕ~=1K0∫0ϕK(τ)dτ,

(12) θ~=1K0∫0θK(τ)dτ.

Differentiating (11) with respect to *x*_*i*_, we get

(13) $$\begin{array}{c} K_{0}{\tilde{\phi }}_{,i}=K\left(\phi \right)\phi_{,i}. \end{array}$$

Differentiating again the above equation, we obtain

(14) $$\begin{array}{c} K_{0}{\tilde{\phi }}_{,ii}={\left[K\left(\phi \right)\phi_{,i}\right]}_{,i}. \end{array}$$

Differentiating (12) with respect to *x*_*i*_, we get

(15) $$\begin{array}{c} K_{0}{\tilde{\theta }}_{,i}=K\left(\theta \right)\theta_{,i}. \end{array}$$

Differentiating (12) with respect to *t*, we get

(16) $$\begin{array}{c} K_{0}\dot{\tilde{\theta }}=K\left(\theta \right)\dot{\theta }. \end{array}$$

Substituting from (14) and (16) into (7), we obtain

(17) Iξ−1(1+τT∂∂t+τT22∂2∂t2)ϕ~,ii =(1+τq∂∂t+τq22∂2∂t2)(θ~˙κ+γT0K0e˙)  +aK0K12[Iξ−1(1+τT∂∂t+τT22∂2∂t2)(ϕ,i)2].

Neglecting the last term on right hand side of the above equation due to nonlinearity, we get

(18) Iξ−1(1+τT∂∂t+τT22∂2∂t2)ϕ~,ii =(1+τq∂∂t+τq22∂2∂t2)(θ~˙κ+γT0K0e˙).

Using (15) in (6), we get

(19) $$\begin{array}{c} \rho {\ddot{u}}_{i}=\left(\lambda +\mu \right)u_{j,ji}+\mu u_{i,jj}-\frac{\gamma K_{0}}{K\left(\theta \right)}{\tilde{\theta }}_{,i}. \end{array}$$

For linearity we can approximate last equation to the following form:

(20) $$\begin{array}{c} \rho {\ddot{u}}_{i}=\left(\lambda +\mu \right)u_{j,ji}+\mu u_{i,jj}-\gamma {\tilde{\theta }}_{,i}. \end{array}$$

Now to transform (8) by using (11) and (12), we first replace the dummy variable *i* with *k* and then differentiating with respect to *x*_*i*_ and finally multiplying by *K*(*θ*) we get

(21) $$\begin{array}{c} K\left(\theta \right)\phi_{,i}-K\left(\theta \right)\theta_{,i}=aK\left(\theta \right)\phi_{,kki},\;i,k=1,2,3. \end{array}$$

Now substituting from (10) into (21) we have

(22) K(ϕ)ϕ,i−K(θ)θ,i =aK(ϕ)ϕ,kki+aK0K12[{(ϕ,i)2},i−a{(ϕ,kk)2},i].

For linearity we can approximate the last equation as

(23) $$\begin{array}{c} K\left(\phi \right)\phi_{,i}-K\left(\theta \right)\theta_{,i}=aK\left(\phi \right)\phi_{,kki}. \end{array}$$

Retaining only the linear terms, (14) can be written as

(24) $$\begin{array}{c} K_{0}{\tilde{\phi }}_{,kki}=K\left(\phi \right)\phi_{,kki}. \end{array}$$

Now substituting from (13), (15), and (24) into (23) we have

(25) $$\begin{array}{c} {\tilde{\phi }}_{,i}-{\tilde{\theta }}_{,i}=a{\tilde{\phi }}_{,kki},\;i,k=1,2,3. \end{array}$$

Now integrating with respect to *x*_*i*_ we get

(26) $$\begin{array}{c} \tilde{\phi }-\tilde{\theta }=a{\tilde{\phi }}_{,ii},\;i=1,2,3. \end{array}$$

## 4. Formulation of the Problem

We consider a half space (0 ≤ *x* < *∞*) with *x*-axis pointing to the medium. This half-space is subjected to thermal and mechanical loads on the bounding plane (*x* = 0) that depends on the time *t* and is linearly quiescent. We will consider one-dimensional thermoelastic deformation of the body so that the displacement components can be taken in the following form:

(27) $$\begin{array}{c} \left(u_{x},u_{y},u_{z}\right)=\left(u\left(x,t\right),0,0\right). \end{array}$$

The strain displacement relation is

(28) $$\begin{array}{c} e_{xx}=\frac{\partial u}{\partial x} \end{array}$$

and the constitutive relation (5) takes the form

(29) $$\begin{array}{c} \sigma_{xx}=\left(\lambda +2\mu \right)\frac{\partial u}{\partial x}-\gamma \theta . \end{array}$$

The equation of motion, heat transport equation, and relation between conductive temperature and thermodynamic temperature can be written as

(30) ρu¨=(λ+2μ)∂2u∂x2−γ∂θ~∂x,Iξ−1(1+τT∂∂t+τT22∂2∂t2)∂2ϕ~∂x2 =(1+τq∂∂t+τq22∂2∂t2)(θ~˙κ+γT0K0e˙),ϕ~−θ~=a∂2ϕ~∂x2.

We now use the following nondimensional variables, to make the above equations nondimensional:

(31) x′=c0κx,  u′=c0κu,  t′=c02κt,τT′=c02κτT,  τq′=c02κτq,  σxx′=σxxλ+2μ,ϕ′=ϕT0,  ϕ~′=ϕ~T0,  θ′=θT0,θ~′=θ~T0,  c02=λ+2μρ.

Then the corresponding nondimensional equations, after omitting the primes, are

(32) σxx=∂u∂x−ϵ2θ,u¨=∂2u∂x2−ϵ2∂θ~∂x,Iξ−1(1+τT∂∂t+τT22∂2∂t2)∂2ϕ~∂x2 =(1+τq∂∂t+τq22∂2∂t2)(θ~˙+ϵ1e˙),ϕ~−θ~=β∂2ϕ~∂x2,

where

(33) $$\begin{array}{c} \epsilon_{1}=\frac{\gamma \kappa }{K_{0}},\;\;\epsilon_{2}=\frac{\gamma T_{0}}{\left(\lambda +2\mu \right)},\;\;\beta =\frac{ac_{0}^{2}}{\kappa^{2}}. \end{array}$$

Initial and regularity conditions for the problem are given by

(34) $$\begin{array}{c} u=\theta =\phi =0\;\text{at}\;t=0\;\text{for}\;x\geq 0,\\ \frac{\partial u}{\partial t}=\frac{\partial \theta }{\partial t}=\frac{\partial \phi }{\partial t}=0\;\text{at}\;t=0\;\text{for}\;x\geq 0,\\ u=\theta =\phi =0\;\text{as}\;x⟶\infty . \end{array}$$

## 5. Method of Solution

Applying the Laplace transform defined by

(35) f−(s)=∫0∞e−stf(t)dt, Re(s)>0

to both the sides of (32), we obtain

(36) $$\begin{array}{c} {\overset{-}{\sigma }}_{xx}=\overset{-}{e}-\epsilon_{2}\overset{-}{\theta }, \end{array}$$

(37) $$\begin{array}{c} \frac{d^{2}\overset{-}{e}}{dx^{2}}=s^{2}\overset{-}{e}+\epsilon_{2}\frac{d^{2}\overset{-}{\tilde{\theta }}}{dx^{2}}, \end{array}$$

(38) (1+τTs+12τT2s2)d2ϕ~−dx2 =sξ(1+τqs+12τq2s2)(θ~−+ϵ1e−),

(39) $$\begin{array}{c} \overset{-}{\tilde{\phi }}-\overset{-}{\tilde{\theta }}=\beta \frac{d^{2}\overset{-}{\tilde{\phi }}}{dx^{2}}. \end{array}$$

Eliminating $\overset{-}{\tilde{\theta }}$ from (37)–(39) we get

(40) d2ϕ~−dx2=α1ϕ~−+ϵ1α1e−,d2e−dx2=α2ϕ~−+α3e−,

where

(41) a=(1+τqs+(1/2)τq2s21+τTs+(1/2)τT2s2),α1=asξ1+aβsξ,  α2=α1ϵ2(1−βα1)[1+βα1ϵ1ϵ2],α3=s2+α1ϵ1ϵ2(1−βα1)[1+βα1ϵ1ϵ2].

### 5.1. State Space Approach

The equations (40) can be written in the form of a vector matrix differential equations [60] as follows:

(42) $$\begin{array}{c} \frac{d^{2}\overset{-}{V}\left(x,s\right)}{dx^{2}}=A\left(s\right)\overset{-}{V}\left(x,s\right), \end{array}$$

where

(43) $$\begin{array}{c} \overset{-}{V}\left(x,s\right)=\left[\begin{array}{c} \overset{-}{\tilde{\phi }}\left(x,s\right)\\ \overset{-}{e}\left(x,s\right) \end{array}\right],\;\;A\left(s\right)=\left[\begin{array}{cc} \alpha_{1} & \epsilon_{1}\alpha_{1}\\ \alpha_{2} & \alpha_{3} \end{array}\right]. \end{array}$$

The formal solution of system (42) bounded at infinity can be written as

(44) $$\begin{array}{c} \overset{-}{V}\left(x,s\right)=\exp\left[-\sqrt{A\left(s\right)}x\right]\overset{-}{V}\left(0,s\right), \end{array}$$

where

(45) $$\begin{array}{c} \overset{-}{V}\left(0,s\right)=\left[\begin{array}{c} \overset{-}{\tilde{\phi }}\left(0,s\right)\\ \overset{-}{e}\left(0,s\right) \end{array}\right]=\left[\begin{array}{c} {\overset{-}{\tilde{\phi }}}_{0}\\ {\overset{-}{e}}_{0} \end{array}\right],\\ \phi_{0}=\phi \left(0,t\right),\;\;e_{0}=e\left(0,t\right). \end{array}$$

We will use the well-known Cayley-Hamilton theorem to find the form of the matrix

(46) $$\begin{array}{c} \exp\left[-\sqrt{A\left(s\right)}x\right]. \end{array}$$

The characteristic equation for the matrix *A*(*s*) can be written as

(47) $$\begin{array}{c} k^{2}-k\left(\alpha_{1}+\alpha_{3}\right)+\left(\alpha_{1}\alpha_{3}-\epsilon_{1}\alpha_{1}\alpha_{2}\right)=0. \end{array}$$

The roots of this equation, namely, *k*_1_ and *k*_2_, satisfy the following relations:

(48) $$\begin{array}{c} k_{1}+k_{2}=\alpha_{1}+\alpha_{3},\\ k_{1}k_{2}=\alpha_{1}\alpha_{3}-\epsilon_{1}\alpha_{1}\alpha_{2}. \end{array}$$

The Taylor series expansion for the matrix exponential in (44) is given by

(49) $$\begin{array}{c} \exp\left[-\sqrt{A\left(s\right)}x\right]=\overset{\infty }{\underset{n=0}{\sum }}\frac{{\left[-\sqrt{A(s)}x\right]}^{n}}{n!}. \end{array}$$

Using Cayley-Hamilton theorem, we can express *A* ^2^ and higher powers of the matrix *A* in terms of *I* and *A*, where *I* is the unit matrix of the second order.

Thus the infinite series in (49) can be reduced to the form

(50) $$\begin{array}{c} \exp\left[-\sqrt{A\left(s\right)}x\right]=a_{0}\left(x,s\right)I+a_{1}\left(x,s\right)A\left(s\right), \end{array}$$

where *a*_0_ and *a*_1_ are coefficients depending on *s* and *x*.

By Cayley-Hamilton theorem, the characteristic roots *k*_1_ and *k*_2_ of the matrix *A* must satisfy (50); thus we have

(51) exp⁡(−k1x)=a0+a1k1,exp⁡(−k2x)=a0+a1k2.

By solving the above linear system of equations, we get

(52) a0=k1e−k2x−k2e−k1xk1−k2,a1=e−k1x−e−k2xk1−k2.

Hence from (50) we get

(53) $$\begin{array}{c} \exp\left[-\sqrt{A\left(s\right)}x\right]=L_{ij}\left(x,s\right),\;i,j=1,2, \end{array}$$

where

(54) L11=e−k2x(k1−α1)−e−k1x(k2−α1)k1−k2,L12=ϵ1α1(e−k1x−e−k2x)k1−k2,L21=α2(e−k1x−e−k2x)k1−k2,L22=e−k1x(α3−k2)−e−k2x(α3−k1)k1−k2.

Using (53) we can write the solution in (44) in the following form:

(55) $$\begin{array}{c} \overset{-}{V}\left(x,s\right)=L_{ij}\left(x,s\right)\overset{-}{V}\left(0,s\right). \end{array}$$

Hence the solution for $\overset{-}{\tilde{\phi }}(x,s)$ and $\overset{-}{e}(x,s)$ can be obtained from (55) as follows:

(56) ϕ~−(x,s)=1k1−k2[e−k1x{ϵ1α1e−0−(k2−α1)ϕ~−0}mmmmmm− e−k2x{ϵ1α1e−0−(k1−α1)ϕ~−0}],

(57) e−(x,s)=1k1−k2[e−k1x{α2ϕ~−0−(k2−α3)e−0}mmmmmm− e−k2x{α2ϕ~−0−(k1−α3)e−0}].

Using (56), the solution for $\overset{-}{\tilde{\theta }}$ can be obtained from (39) as follows:

(58) θ~−=1k1−k2[e−k1x{ϵ1α1e−0−(k2−α1)ϕ~−0}(1−βk1)mmmmmm−e−k2x{ϵ1α1e−0−(k1−α1)ϕ~−0}(1−βk2)].

### 5.2. Application to Thermal Shock Problem

We will consider the bounding plane of the medium at *x* = 0 subjected to thermal shock in the following nondimensional form:

(59) $$\begin{array}{c} \phi \left(0,t\right)=\phi_{1}H\left(t\right), \end{array}$$

where *ϕ*_1_ is constant. Now applying the Laplace transform to (59) we get

(60) $$\begin{array}{c} \overset{-}{\phi }\left(0,s\right)=\frac{\phi_{1}}{s}. \end{array}$$

Using (9), (11), and (60) we get

(61) $$\begin{array}{c} \overset{-}{\tilde{\phi }}\left(0,s\right)=\frac{l}{s}, \end{array}$$

where *l* = *ϕ*_1_ + (*K*_1_/2*s*)*ϕ*_1_^2^.

### 5.3. The Mechanical Boundary Condition

The mechanical boundary condition is taken in the form

(62) $$\begin{array}{c} e\left(0,t\right)=0. \end{array}$$

This implies

(63) $$\begin{array}{c} \overset{-}{e}\left(0,s\right)={\overset{-}{e}}_{0}=0. \end{array}$$

Applying the boundary conditions (61) and (63) to (56)–(58) we get

(64) ϕ~−=ls(k1−k2)[(α1−k2)e−k1x−(α1−k1)e−k2x],

(65) e−=lα2s(k1−k2)[e−k1x−e−k2x],

(66) θ~−=ls(k1−k2)[(α1−k2)(1−βk1)e−k1x    mmmimmm−(α1−k1)(1−βk2)e−k2x].

Displacement component $\overset{-}{u}$ can be obtained from (28) using (65) in the following form:

(67) $$\begin{array}{c} \overset{-}{u}=\frac{-l\alpha_{2}}{s\left(k_{1}-k_{2}\right)}\left[\frac{e^{-\sqrt{k_{1}}x}}{{\sqrt{k}}_{1}}-\frac{e^{-\sqrt{k_{2}}x}}{{\sqrt{k}}_{2}}\right]. \end{array}$$

Using (9), (11), and (64) the solution for $\overset{-}{\phi }$ can be obtained as follows:

(68) ϕ−=1K1[−(α1−k1)e−k2x]2K1ls(k1−k2))1/2(1+2K1ls(k1−k2)mmmnmm×[(α1−k2)e−k1xmmmmmmm−(α1−k1)e−k2x]2K1ls(k1−k2))1/2−1], K1<0ϕ−=ls(k1−k2)[(α1−k2)e−k1xmmmmmmmm−(α1−k1)e−k2x], K1=0.

Again using (9), (12), and (66) the solution for $\overset{-}{\theta }$ can be obtained in the following form:

(69) θ−=ls(k1−k2)[(α1−k2)(1−βk1)e−k1xmmmmmmmm−(α1−k1)(1−βk2)e−k2x]θ−=θ~0(say), K1=0θ−=1K1[1+2K1θ~0−1], K1<0.

The solution for ${\overset{-}{\sigma }}_{xx}$ can be obtained from (36) using (65) and (69) in the following form:

(70) σ−xx=lα2s(k1−k2)[e−k1x−e−k2x]σ−xx −ϵ2K1[1+2K1θ~0−1], K1<0;σ−xx=lα2s(k1−k2)[e−k1x−e−k2x]−ϵ2θ~0,mmmmmmmmmmmmmmnmmmK1=0.

This completes the solution of the thermal shock problem in Laplace transform domain.

## 6. Numerical Inversion of Laplace Transform

It is difficult to find the analytical inverse of Laplace transform of the complicated solutions for the displacement, thermodynamic temperature, conductive temperature, stress, and strain in Laplace transform domain. So we have to resort to numerical computations. We now outline the numerical procedure to solve the problem. Let $\overset{-}{f}(x,s)$ be the Laplace transform of a function *f*(*x*, *t*).

Then the inversion formula for Laplace transform can be written as

(71) $$\begin{array}{c} f\left(x,t\right)=\frac{1}{2\pi i}\overset{d+i\infty }{\underset{d-i\infty }{\int }}e^{st}\overset{-}{f}\left(x,s\right)ds, \end{array}$$

where *d* is an arbitrary real number greater than real parts of all the singularities of $\overset{-}{f}(x,s)$.

Taking *s* = *d* + *iw*, the preceding integral takes the form

(72) $$\begin{array}{c} f\left(x,t\right)=\frac{e^{dt}}{2\pi }\overset{\infty }{\underset{-\infty }{\int }}e^{itw}f\left(x,d+iw\right)dw. \end{array}$$

Expanding the function *h*(*x*, *t*) = *e* ^−*dt*^ *f*(*x*, *t*) in a Fourier series in the interval [0,2*T*] we obtain the approximate formula [32]

(73) $$\begin{array}{c} f\left(x,t\right)=f_{\infty }\left(x,t\right)+E_{D}, \end{array}$$

where

(74) $$\begin{array}{c} f_{\infty }\left(x,t\right)=\frac{1}{2}c_{0}+\overset{\infty }{\underset{k=1}{\sum }}c_{k}\;\text{for}\;0\leq t\leq 2T,\\ c_{k}=\frac{e^{dt}}{T}\left[e^{ik\pi t/T}\;\overset{-}{f}\left(x,d+\frac{ik\pi t}{T}\right)\right]. \end{array}$$

The discretization error *E*_*D*_ can be made arbitrary small by choosing *d* large enough [32]. Since the infinite series in (74) can be summed up to a finite number *N* of terms, the approximate value of *f*(*x*, *t*) becomes

(75) $$\begin{array}{c} f_{N}\left(x,t\right)=\frac{1}{2}c_{0}+\overset{N}{\underset{k=1}{\sum }}c_{k}\;\text{for}\;0\leq t\leq 2T. \end{array}$$

Using the preceding formula to evaluate *f*(*x*, *t*) we introduce a truncation error *E*_*T*_ that must be added to the discretization error to produce total approximation error.

Two methods are used to reduce the total error. First the “Korrektur” method is used to reduce the discretization error. Next the *ε*-algorithm is used to accelerate convergence [32].

The Korrektur method uses the following formula to evaluate the function *f*(*x*, *t*):

(76) $$\begin{array}{c} f\left(x,t\right)=f_{\infty }\left(x,t\right)-e^{-2dT}f_{\infty }\left(x,2T+t\right)+E_{D}^{\prime }, \end{array}$$

where the discretization error |*E*_*D*_′ | ≪|*E*_*D*_|. Thus, the approximate value of *f*(*x*, *t*) becomes

(77) $$\begin{array}{c} f_{NK}\left(x,t\right)=f_{N}\left(x,t\right)-e^{-2dT}f_{N^{\prime }}\left(x,2T+t\right), \end{array}$$

where *N*′ is an integer such that *N*′ < *N*.

We will now describe the *ε*-algorithm that is used to accelerate the convergence of the series in (75). Let *N* = 2*q* + 1, where *q* is a natural number, and let *s*_*m*_ = ∑_*k*=1_^*m*^ *c*_*k*_ be the sequence of partial sum of series in (75).

We define the *ε*-sequence by

(78) $$\begin{array}{c} \varepsilon_{0,m}=0,\;\;\varepsilon_{1,m}=s_{m},\\ \varepsilon_{p+1,m}=\varepsilon_{p-1,m+1}+\frac{1}{\varepsilon_{p,m+1}-\varepsilon_{p,m}},\;p=1,2,3,\ldots . \end{array}$$

It can be shown that [32] the sequence

(79) $$\begin{array}{c} \varepsilon_{1,1},\varepsilon_{3,1},\varepsilon_{5,1},\ldots ,\varepsilon_{N,1} \end{array}$$

converges to *f*(*x*, *t*) + *E*_*D*_ − (*c*_0_/2) faster than the sequence of partial sums *s*_*m*_, *m* = 1,2, 3,….

The actual procedure used to invert the Laplace transform consists of using (77) together with the *ε*-algorithm. The values of *d* and *T* are chosen according to the criterion outlined in [32].

## 7. Numerical Results and Discussion

To get the solution for strain (*e*), thermal displacement component (*u*), conductive temperature (*ϕ*), thermodynamic temperature (*θ*), and thermal stress (*σ*_*xx*_) in the space time domain we have applied Laplace inversion formula to (65), (67), (68), (69), and (70), respectively, which have been done numerically using a method based on Fourier series expansion technique [32]. The numerical code has been prepared using Fortran 77 programming language. For computational purpose copper material has been taken into consideration. The values of the material constants are taken as follows [61]:

(80) λ=7.76×1010 Nm−2,μ=3.86×1010 Nm−2,ρ=8954 Kg m−3,K0=386 Wm−1 K−1,cE=383.1 JKg−1 K−1,  T0=293 K,αt=1.78×10−5 K−1,  ε1=1.618,ε2=0.01041,  β=0.1,τq=0.02,  τT=0.015.

Also we take time *t* = 0.2, *ϕ*_1_ = 1 for computational purpose.

Figures 1, 2, 3, 4, 5, 6, 7, 8, 9, 10, 11, 12, 13, 14, and 15 are drawn to represent the variation of said thermophysical quantities versus the space variable *x* for different *K*_1_ (=0,−2,−4), *ξ* (=0.5,1.0,1.6), and *β* (=0.0,0.1). Here *ξ* = 0.5, 1.0, and 1.6 corresponds to week conductivity, normal conductivity, and super conductivity, respectively; *β* = 0.0 and 0.1 corresponds to one-temperature and two-temperature theory, respectively.

**Figure 1:**
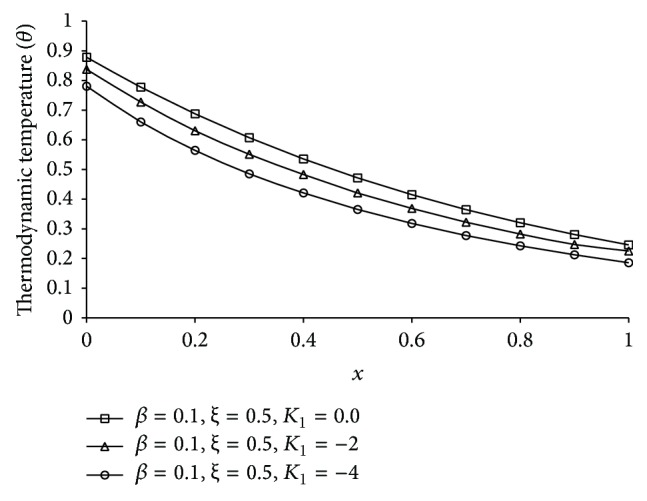
The thermodynamic temperature (*θ*) for different *K*_1_ at *t* = 0.2.

**Figure 2 fig2:**
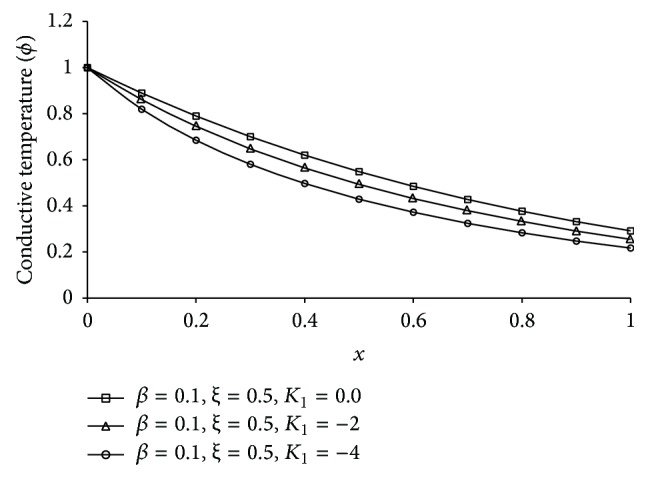
The conductive temperature (*ϕ*) for different *K*_1_ at *t* = 0.2.

**Figure 3 fig3:**
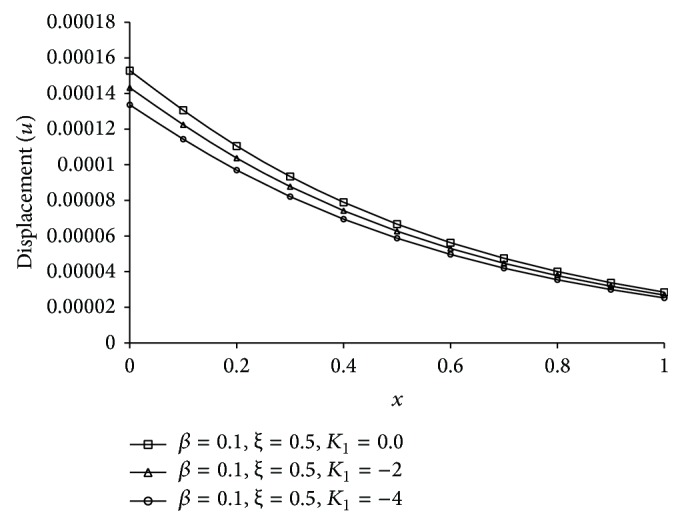
The displacement (*u*) for different *K*_1_ at *t* = 0.2.

**Figure 4 fig4:**
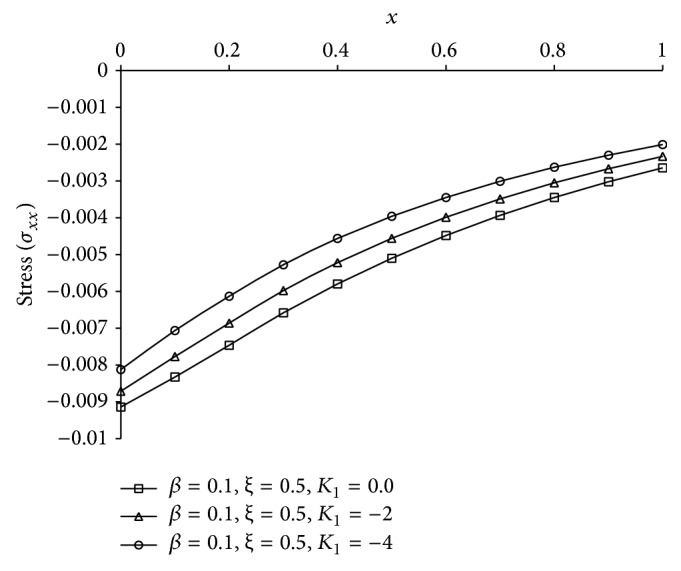
The stress (*σ*_*xx*_) for different *K*_1_ at *t* = 0.2.

**Figure 5 fig5:**
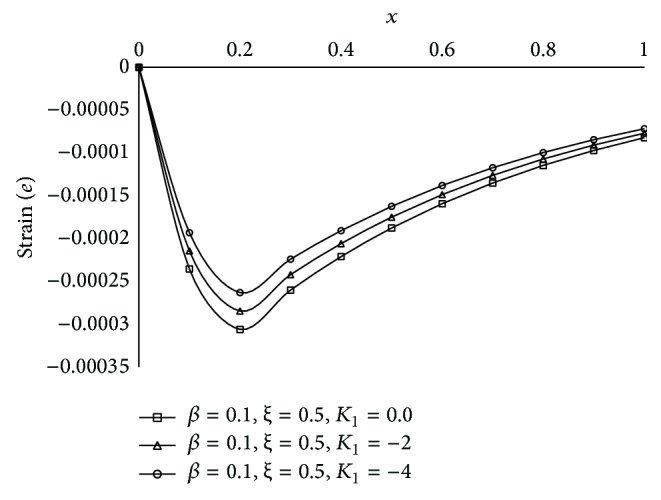
The strain (*e*) for different *K*_1_ at *t* = 0.2.

Figures 1 [Fig fig2] [Fig fig3] [Fig fig4]–5 show the effect of *K*_1_ on the said five thermophysical quantities for two-temperature theory (*β* = 0.1) and fractional order parameter *ξ* = 0.5. From these figures it is clear that magnitude of all the quantities, that is, thermodynamic temperature *θ*, conductive temperature *ϕ*, displacement *u*, stress component *σ*, and strain component *e*, is greater for smaller magnitude of *K*_1_.

**Figure 6 fig6:**
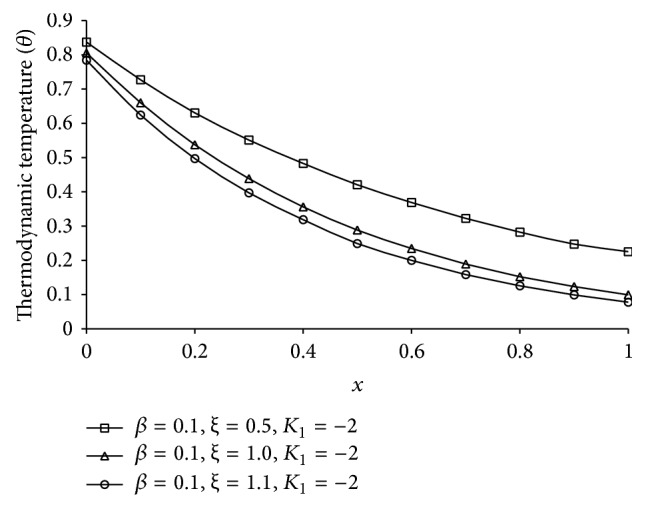
The thermodynamic temperature (*θ*) for different *ξ* at *t* = 0.2.

**Figure 7 fig7:**
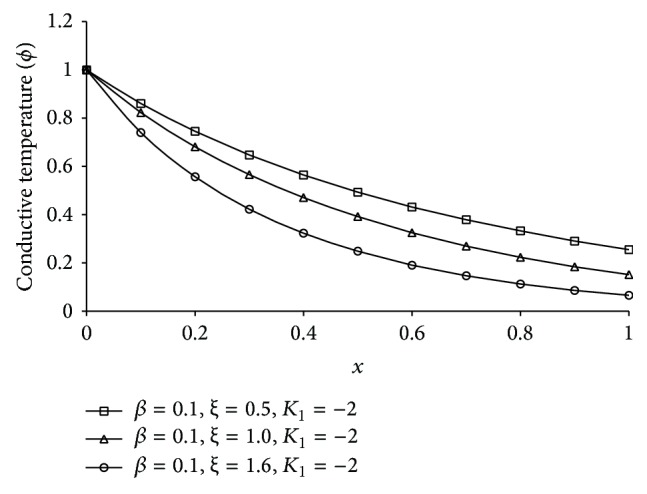
The conductive temperature (*ϕ*) for different *ξ* at *t* = 0.2.

**Figure 8 fig8:**
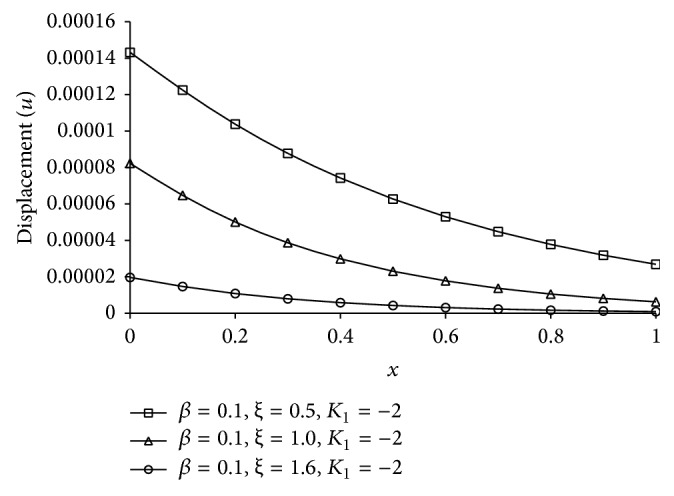
The displacement (*u*) for different *ξ* at *t* = 0.2.

**Figure 9 fig9:**
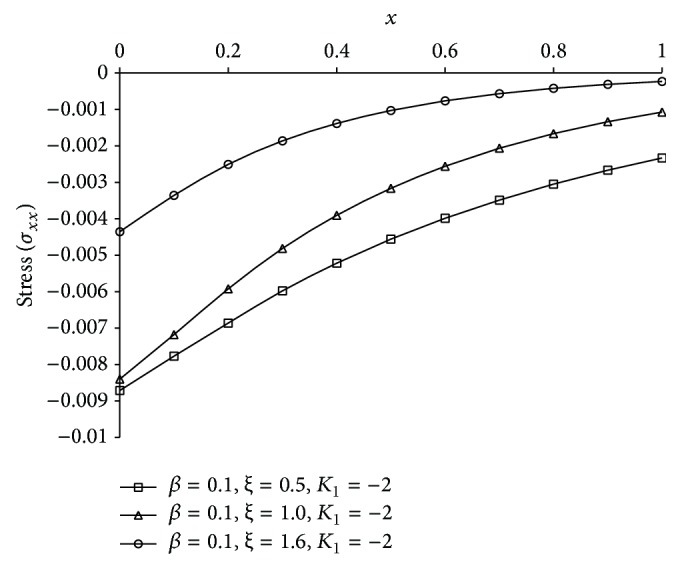
The stress (*σ*_*xx*_) for different *ξ* at *t* = 0.2.

**Figure 10 fig10:**
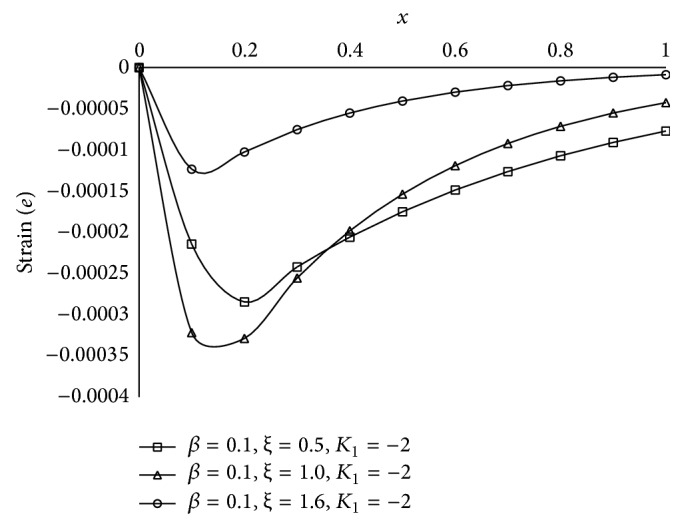
The strain (*e*) for different *ξ* at *t* = 0.2.

Figures 6 [Fig fig7] [Fig fig8] [Fig fig9]–10 show the effect of *ξ* for two-temperature (*β* = 0.1) theory and *K*_1_ = −2 on those five quantities. Figures 6 [Fig fig7] [Fig fig8]–9 show that the magnitude of thermodynamic temperature *θ*, conductive temperature *ϕ*, displacement component *u*, and stress component *σ* has greater value for smaller magnitude of *ξ*. But from Figure 10 it is observed that in the region 0.0 ≤ *x* < 0.3 (approximate) strain component has larger value for *ξ* = 1.0 than for *ξ* = 0.5, which is again larger than for *ξ* = 1.6.

**Figure 11 fig11:**
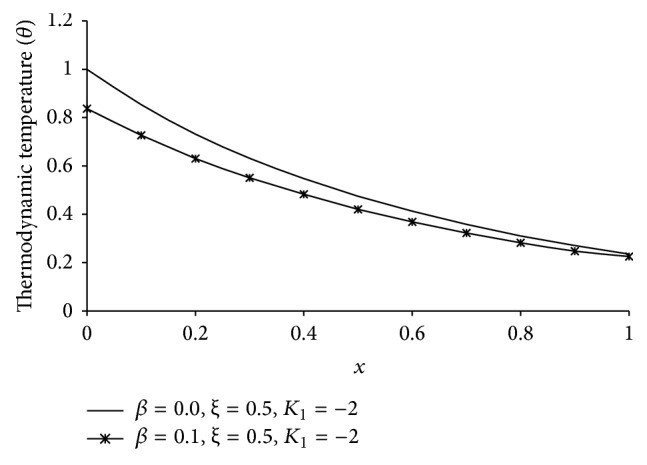
The comparison of thermodynamic temperature (*θ*) between 1TT and 2TT at *t* = 0.2.

**Figure 12 fig12:**
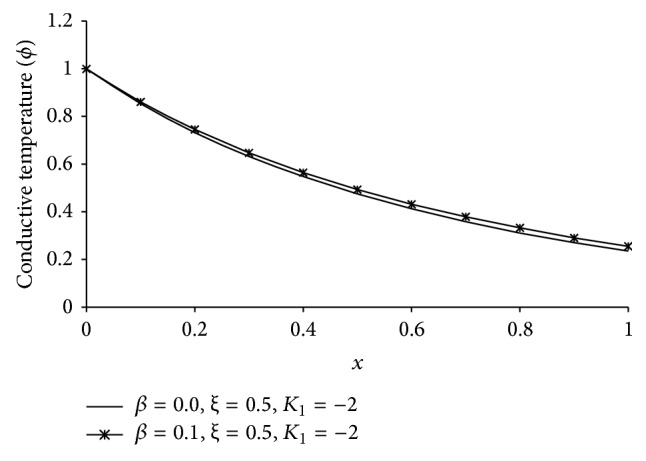
The comparison of conductive temperature (*ϕ*) between 1TT and 2TT at *t* = 0.2.

**Figure 13 fig13:**
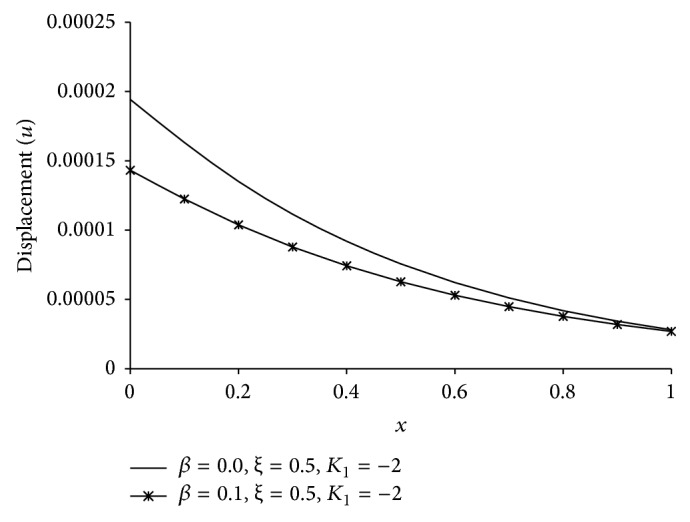
The comparison of displacement (*u*) between 1TT and 2TT at *t* = 0.2.

**Figure 14 fig14:**
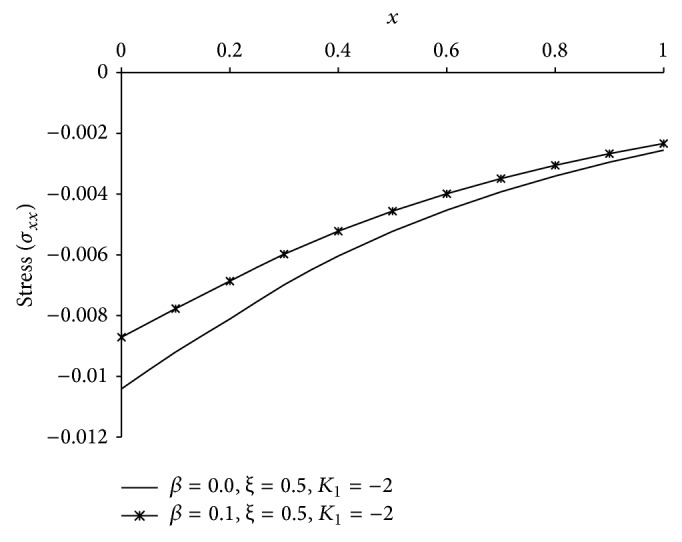
The comparison of stress (*σ*_*xx*_) between 1TT and 2TT at *t* = 0.2.

**Figure 15:**
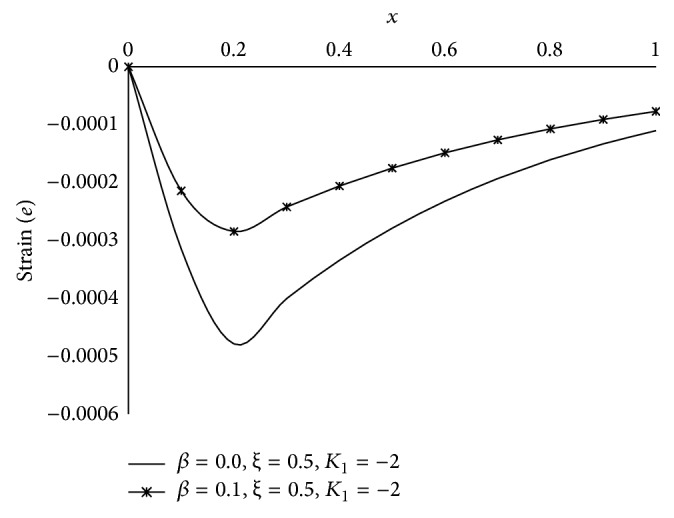
The comparison of radial strain (*e*) between 1TT and 2TT at *t* = 0.2.

Figures 11 [Fig fig12] [Fig fig13] [Fig fig14]–15 are drawn to compare between the results of one-temperature (*β* = 0.0) theory and two-temperature (*β* = 0.1) theory for *ξ* = 0.5 and *K*_1_ = −2 for five different thermophysical quantities. Figures 11, 13, 14, and 15 show that the magnitude of *θ*, *u*, *σ*, *e* is greater for one-temperature (*β* = 0.0) case than two-temperature (*β* = 0.1) case. The only exception is *ϕ* here.

It is observed that at the boundary plane *x* = 0, *ϕ* = 1 (Figures 2 [Fig fig3] [Fig fig4] [Fig fig5] [Fig fig6], 7 [Fig fig8] [Fig fig9] [Fig fig10] [Fig fig11], and 12), and *e* = 0 (Figures 5 [Fig fig6] [Fig fig7] [Fig fig8] [Fig fig9], 10, and 15), which satisfies our theoretical boundary condition. It ensures the correctness of the numerical code used. In Figures 6 [Fig fig7] [Fig fig8] [Fig fig9]–10 the results for *ξ* = 1 agree with the corresponding results of Mondal et al. [24].

**Figure 16:**
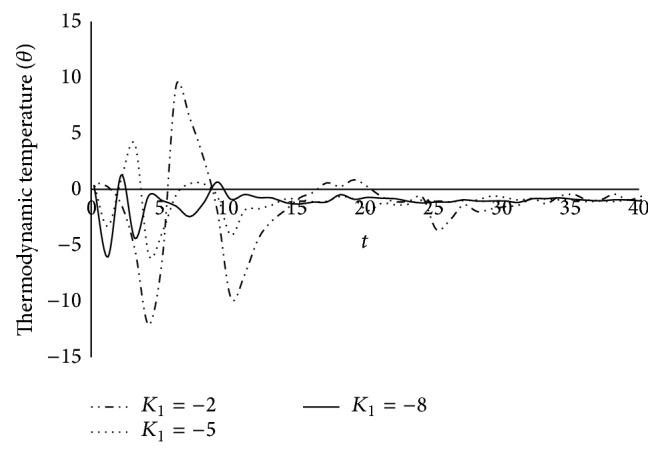
The variation of thermodynamic temperature (*θ*) with time (*t*) at *x* = 0.2 for *β* = 0.1; *ξ* = 0.5.

**Figure 17:**
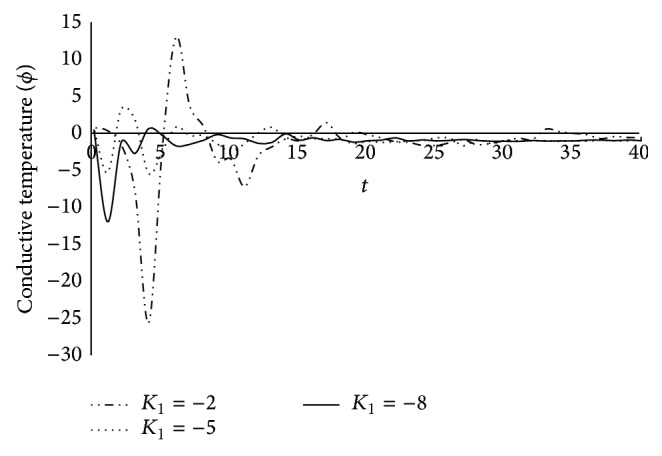
The variation of conductive temperature (*ϕ*) with time (*t*) at *x* = 0.2 for *β* = 0.1; *ξ* = 0.5.

Figures 16 and 17 represent the variation of the thermodynamic temperature *θ* and conductive temperature *ϕ* against time *t* for different value of *K*_1_, namely, *K*_1_ = −2, −5, −8, when *β* = 0.1, *ξ* = 0.5, and *x* = 0.2. It is observed from the figures that the magnitudes of *θ* and *ϕ* are oscillatory in nature and the magnitude of peek of oscillation decreases with time.

## 8. Conclusion

State space approach has been applied to solve a generalized thermoelastic problem of an isotropic, half-space with variable thermal conductivity. The boundary (*x* = 0) of the half-space is subjected to thermal and mechanical loads. Variation of thermal conductivity has been taken as linear function of temperature. The problem has been studied using the two-temperataure dual-phase-lag model of generalized thermoelasticity in consideration of fractional order heat conduction equation.

(1) The phenomenon of finite speeds of propagation is observed in all depicted figures. This is expected since the thermal wave travels with finite speed.
(2) The effects of the fractional parameter on all the studied fields are very significant.
(3) The value of *K*_1_ has an essential role in changing the value of the distributions.
(4) Significant differences in the physical quantities are observed between the one-temperature theory and the two-temperature theory. The two-temperature theory is more realistic than the one-temperature theory in the case of generalized thermoelasticity.
(5) Initially (at *t* = 0) the conductive temperature has the value of 1 and strain has the value of 0. These results agree with the boundary conditions.

## Nomenclature

*λ*, *μ*: Lamé's constant
*ρ*: Density
*c*_*E*_: Specific heat at constant strain
*t*: Time
*ξ*: Parameter of the Riemann-Liouville fractional integral
*ϕ*: Conductive temperature
*θ*: Thermodynamic temperature
*α*_*t*_: Coefficient of linear thermal expansion
*σ*_*ij*_: Components of stress tensor
*e*_*ij*_: Components of strain tensor
*u*_*i*_: Components of displacement vector
*K*: Thermal conductivity
*τ*_0_: Relaxation time
$c_{0}=\sqrt{(\lambda +2\mu )/\rho }$: Longitudinal wave speed
*κ* = *K*/*ρc*_*E*_: Thermal diffusivity
*a*: Two-temperature parameter
*β* = *ac*_0_^2^/*κ*^2^: Dimensionless two-temperature parameter
*ϵ*_2_ = *γT*_0_/(*λ* + 2*μ*): Dimensionless mechanical coupling constant
$\vec{q}$: Heat flux vector
*ν* = *λ*/2(*λ* + *μ*): Poisson's ratio.
